# Encapsulation of *Nigella sativa* Essential Oil by Complex Coacervation Using Gelatin and Gum Arabic

**DOI:** 10.1111/1750-3841.71333

**Published:** 2026-07-31

**Authors:** Neslihan Mutlu, Ümmü Gülsüm Koç

**Affiliations:** ^1^ Department of Biology, Faculty of Science and Letters Kafkas University Kars Türkiye; ^2^ Graduate School of Natural and Applied Sciences Kafkas University Kars Türkiye

## Abstract

This study aimed to encapsulate *Nigella sativa* essential oil (NSEO) using gelatin (Ge) and gum arabic (GA) via complex coacervation to produce NSEO microcapsules (NSEO‐MCs), optimize encapsulation conditions, characterize the microcapsules, and evaluate antimicrobial activity. Response surface methodology (RSM) based on a Box–Behnken design was used to optimize pH, wall material concentration, and oil concentration. Optimal conditions were pH 4.09, wall material concentration 1.52%, and oil concentration 0.64%, yielding a predicted encapsulation efficiency (EE) of 87.38%. Freeze‐dried microcapsules exhibited moisture content (MC) of 4.65%, hygroscopicity of 13.08%, water solubility (WS) of 19.44%, and moderate flowability, with a Carr's index (CI) of 17.78% and Hausner ratio (HR) of 1.22. X‐ray diffraction (XRD) revealed an amorphous character in NSEO‐MCs, with increased diffraction intensity at 2*θ* ≈ 20° attributed to the compact Ge–GA coacervate matrix. Fourier transform infrared spectroscopy (FTIR) confirmed successful encapsulation through attenuation of NSEO‐characteristic C─H stretching bands and Amide I profile changes, indicating noncovalent interactions between Ge and GA. Thermogravimetric analysis (TGA) demonstrated enhanced thermal stability, with the derivative thermogravimetry (DTG) peak shifting to higher temperatures and broadening relative to free NSEO, confirming the protective role of the wall matrix. Antimicrobial evaluation by agar well diffusion showed dose‐dependent inhibitory activity of both NSEO‐MCs and free NSEO against *Escherichia coli* and *Staphylococcus aureus*. No significant difference was observed between NSEO‐MCs and free NSEO at any tested concentration, confirming that encapsulation preserved antimicrobial efficacy. These results indicate that Ge–GA complex coacervation effectively protects NSEO while retaining its bioactive properties.

## 1 Introduction

*Nigella sativa* L., a member of the Ranunculaceae family, is an annual plant widely known as black cumin. Its seeds are valued for their rich nutritional composition, containing carbohydrates, proteins, essential amino acids, vitamins, and various minerals. They also contain about 30%–40% fixed oil, which is mainly composed of unsaturated fatty acids such as linoleic and oleic acids, along with β‐sitosterol. In addition, the seeds produce a volatile oil rich in bioactive compounds, including thymoquinone, dihydrothymoquinone, carvacrol, p‐cymene, thymol, α‐pinene, and β‐pinene, which contribute to their biological properties such as anti‐inflammatory, antioxidant, and antimicrobial activities (Fatmi et al. 2024; Gürel and Çiçek Polat 2024; Perera et al. 2021). However, the application of NSEO in food systems remains limited due to its peppery and bitter taste, poor solubility, and susceptibility to rapid deterioration. Like many essential oils, NSEO is prone to oxidation and degradation during storage, which necessitates careful packaging and storage conditions. In addition, the bioactive compounds present in NSEO exhibit low stability and can be easily affected by environmental factors such as temperature, light, volatility, and hydrophobicity (Alkhatib et al. 2022; Santiworakun et al. 2022). In this context, microencapsulation has emerged as a promising strategy to overcome these limitations, enabling the controlled incorporation of essential oils into food systems, including active food packaging applications (Qi et al. 2026).

Encapsulation is a process in which active compounds are entrapped within a coating material, enabling controlled release under specific conditions (Alu'datt et al. 2022). Among various encapsulation techniques, microencapsulation by complex coacervation has been widely recognized for its ability to effectively protect and retain unstable ingredients by improving the structural integrity and stability of the resulting microcapsules, as well as for its practical advantages such as simplicity, reproducibility, low cost, and scalability (Kaushik et al. 2015; Sun et al. 2025). This technique relies on electrostatic interactions between oppositely charged biopolymers, leading to the formation of a protective wall around the core material upon pH adjustment and enabling improved stability and controlled release (Muhoza et al. 2023; Yammine et al. 2024). At pH values above the pKa of polysaccharides and below the isoelectric point (pI) of proteins, negatively charged polysaccharides interact with positively charged protein residues, forming protein–polysaccharide complexes that can undergo phase separation and generate a coacervate phase capable of entrapping bioactive compounds (Dong et al. 2023; Li et al. 2024).

There is growing interest in studying electrostatic complexation between proteins and polysaccharides, and complex coacervation systems involving plant‐ and animal‐derived proteins with various polysaccharides have been extensively investigated. These complex coacervates exhibit desirable technological properties, including improved gelling, stabilizing, emulsifying, and foaming capacities, and are widely used for the delivery of bioactive compounds in the nutraceutical industry (Li et al. 2024).

In complex coacervation, Ge (NH^3+^) and GA (COO^−^) are widely used as oppositely charged polymers and represent one of the most conventional protein–polysaccharide systems for microencapsulation. Ge contributes to the formation of a robust capsule wall, while GA provides stability due to its low viscosity, high solubility, and emulsifying capacity. Therefore, the Ge–GA system is considered a natural, biocompatible, and efficient encapsulation approach (Napiórkowska and Kurek 2022; Wang et al. 2024).

Various drying techniques have been used to obtain powdered microcapsules from coacervates, and freeze‐drying is commonly preferred for thermally sensitive bioactive compounds as it avoids high temperatures that may cause degradation. Although complex coacervation has been widely used for microencapsulation, the formation and stability of coacervates can be significantly affected by parameters such as pH, wall material ratio, and core material concentration (Muhoza et al. 2022, 2023). Therefore, optimizing these parameters is essential to obtain microcapsules with high encapsulation efficiency (EE).

Although microencapsulation of *Nigella sativa* oil has been previously explored using spray drying with maltodextrin‐ or whey protein‐based wall systems (Mohammed et al. 2017), the application of Ge–GA complex coacervation for the encapsulation of NSEO remains largely uninvestigated. Complex coacervation offers several advantages over spray drying for thermally sensitive bioactive compounds, including ambient‐temperature processing, higher EE at lower wall material concentrations, and the formation of a mechanically robust polymeric shell through electrostatic interactions (Muhoza et al. 2022). Furthermore, while Box–Behnken‐based response surface methodology (RSM) optimization has been employed in spray drying studies of NSEO to determine optimal process parameters, a systematic RSM‐based approach for optimizing coacervation conditions—specifically pH, wall material concentration, and oil concentration—in the Ge–GA/NSEO system has not been reported.

The present study was designed with a primary focus on food applications, aiming to develop a natural and biocompatible encapsulation system that can be safely incorporated into food products. Therefore, this study aimed to fill this gap by encapsulating NSEO using Ge–GA complex coacervation and optimizing coacervation conditions via a Box–Behnken design to maximize EE. The resulting freeze‐dried microcapsules were comprehensively characterized in terms of physicochemical properties (morphology, moisture content, hygroscopicity, solubility, flowability), structural integrity (X‐ray diffraction [XRD], Fourier transform infrared spectroscopy [FTIR]), thermal stability (thermogravimetric analysis [TGA]), and antimicrobial activity. It was hypothesized that the Ge–GA complex coacervation system would provide an effective and biocompatible encapsulation matrix for NSEO, improving its stability while preserving its bioactive properties.

## 2 Materials and Methods

### 2.1 Materials

Gelatin (Alfasol, 220 Bloom) and GA (Alfasol) were purchased from Kimbiotek Kimyevi Maddeler San. Tic. A.Ş. (Istanbul, Türkiye). NSEO (100% cold‐pressed) was obtained from Hünnap (Istanbul, Türkiye). Mueller–Hinton agar was purchased from Merck (Darmstadt, Germany). *Escherichia coli* ATCC 25922 and *Staphylococcus aureus* ATCC 29213 were obtained from the Microbiology Laboratory, Department of Biology, Kafkas University (Kars, Türkiye).

### 2.2 Preparation of Ge and GA Stock Solutions

Gelatin solution was prepared by dispersing Ge in distilled water and allowing it to hydrate at room temperature for 30 min. The dispersion was then stirred at 600 rpm and 50°C for 2 h using a magnetic stirrer until a homogeneous solution was obtained. Similarly, a GA solution was prepared by gradually adding GA to distilled water under continuous stirring for 2 h to obtain a homogeneous solution.

### 2.3 Optimization of Encapsulation Conditions by RSM

In complex coacervation systems, parameters such as pH, wall material concentration, and oil concentration have been reported as critical factors influencing coacervate formation and the EE of the resulting microcapsules (Chen et al. 2025a; Ma et al. 2024; Plati and Paraskevopoulou 2023).

RSM based on a Box–Behnken design was employed to optimize the encapsulation conditions of NSEO microcapsules (NSEO‐MCs). Three independent variables were investigated; wall material concentration (1%, 1.5%, and 2%), NSEO concentration (0.5%, 1.0%, and 1.5%), and pH (3.5, 4.0, and 4.5). Ge and GA were used as wall materials at a fixed ratio of 1:1. The experimental design was generated using Minitab Statistical Software (version 22, Minitab LLC, State College, PA, USA). The Box–Behnken design consisted of 15 experimental runs, including three replicates at the center point to estimate the experimental error (Table 1). EE were selected as the response variable. The experimental data were fitted to a second‐order polynomial model (Equation 1) to describe the relationship between the independent variables and the response value (Djihad et al. 2024).

(1) $$\begin{array}{ccc} Y & = & \beta_{0}+\beta_{1}A+\beta_{2}B+\beta_{3}C+\beta_{11}A^{2}+\beta_{22}B^{2}+\beta_{33}C^{2}+\beta_{12}AB\\ & & +\beta_{13}AC+\beta_{23}BC \end{array}$$

where *Y* represents the dependent response (EE%); *β*_0_ is the intercept; *β*_1_, *β*_2_, and *β*_3_ are the linear coefficients; *β*_11_, *β*_22_, and *β*_33_ are the quadratic coefficients; and *β*_12_, *β*_13_, and *β*_23_ are the interaction coefficients. *A*, *B*, and *C* represent pH, wall material concentration, and oil concentration, respectively.

**TABLE 1:** Box–Behnken experimental design and experimental values of EE%.

Run	pH	Wall material concentration (%)	NSEO concentration (%)	EE (%)
1	4.5	1.5	0.5	91.70
2	4.0	1.5	1.0	86.80
3	3.5	2.0	1.0	45.00
4	4.5	2.0	1.0	77.20
5	4.0	1.5	1.0	88.40
6	3.5	1.0	1.0	45.00
7	4.0	2.0	1.5	93.60
8	4.0	1.5	1.0	86.72
9	4.0	1.0	0.5	87.73
10	4.5	1.5	1.5	50.00
11	4.0	1.0	1.5	93.92
12	4.5	1.0	1.0	75.00
13	3.5	1.5	0.5	81.50
14	3.5	1.5	1.5	85.46
15	4.0	2.0	0.5	73.36

### 2.4 Encapsulation of NSEO

Microencapsulation by complex coacervation was carried out according to the method described by Hosseini et al., with slight modifications (Hosseini et al. 2013). NSEO was slowly added to the Ge solution and homogenized using a high‐shear homogenizer (DLAB D‐160, DLAB Scientific, China) at 10,000 rpm for 10 min to obtain an oil‐in‐water emulsion. Subsequently, GA solution was added and the mixture was magnetically stirred (IKA Yellowline, IKA‐Werke GmbH & Co. KG, Germany) for 30 min. During stirring, the pH was adjusted to the desired value by adding HCl solution dropwise and monitored using a pH meter (HI2002‐02 Edge, Hanna Instruments, USA) to induce complex coacervation. The formed coacervates were collected by centrifugation at 9000 × *g* for 30 min at 4°C. The collected microcapsules were freeze‐dried at −35°C for 72 h using a freeze dryer (LyoQuest‐85 PLUS ECO, Lyoquest, Telstar, Terrassa, Spain).

### 2.5 Encapsulation Efficiency of NSEO‐MCs

EE of the microcapsules was determined according to the method described by Zhang et al., with slight modifications (Zhang et al. 2023). A capsule sample (0.5 g) was mixed with 50 mL of absolute ethanol and gently shaken to dissolve the surface oil. The mixture was centrifuged (9000 × *g*, 10 min), and the supernatant was collected to determine the free NSEO content by UV–Vis spectrophotometry (Multiskan SkyHigh, Thermo Fisher Scientific, Singapore) at 254 nm (Salmani et al. 2014).

To determine the total NSEO content, 0.5 g of capsule sample was mixed with 50 mL of ethanol and subjected to an ultrasonic bath (Bandelin Electronic RK 255H, Germany) at 25°C with a power of 160 W and a frequency of 35 kHz for 20 min to ensure complete extraction of the oil into the solvent. The extract was filtered and the filtrate was analyzed by UV–Vis spectrophotometry at 254 nm. Absorbance values were converted to concentrations using a calibration curve, and EE (%) was calculated according to Equation (2).

(2) $$\mathrm{EE}\;\left(\%\right)=\frac{\left(\mathrm{Total}\;\mathrm{NSEO}\;-\;\mathrm{Free}\;\mathrm{NSEO}\right)}{\mathrm{Total}\;\mathrm{NSEO}}\;\times \;100$$

where Total NSEO represents the total amount of NSEO in the microcapsules, and Free NSEO represents the amount of surface NSEO.

### 2.6 Characterization of NSEO‐MCs

Microcapsules were prepared under the optimal conditions predicted by the RSM model using the response optimizer, as described in “Encapsulation of NSEO” section, and were then used for subsequent characterization.

#### 2.6.1 Morphology

The morphology of NSEO‐MCs was first observed before freeze‐drying using an optical microscope (Leica DM500, Leica Microsystems, Wetzlar, Germany) at ×100 magnification. After freeze‐drying, the morphology of the microcapsules was examined by scanning electron microscopy (SEM; Gemini 300, Carl Zeiss, Jena, Germany). The samples were mounted on aluminum stubs using double‐sided carbon tape and coated with a thin layer of gold prior to observation. SEM images were obtained at an accelerating voltage of 15 kV with magnifications ranging from ×250 to ×1000.

#### 2.6.2 Hygroscopicity, Moisture Content, and Water Solubility

The hygroscopicity of NSEO‐MCs was determined according to Mohammed et al. (2021). Briefly, 1 g of microcapsule powder was placed in a desiccator containing a saturated NaCl solution (≈75% relative humidity) and stored at room temperature for 7 days. After this period, the samples were weighed and hygroscopicity values were calculated using Equation (4).

Moisture content was determined according to Chen et al. (2025b). Approximately 1 g of microcapsule powder was dried in an oven at 105°C until constant weight was achieved. Moisture content (%) was calculated using Equation (5).

(4) $$\mathrm{Hygroscopicity}\;\;\left(\%\right)=\left(\mathrm{Adsorbed}\;\mathrm{moisture}/\mathrm{Sample}\;\mathrm{weight}\right)\;\times \;100$$

(5) $$\mathrm{Moisture}\;\mathrm{content}\;\;\left(\%\right)=\left(W_{1}\;-\;W_{2}/W_{1}\right)\;\times \;100$$

Here, $W_{1}$ represents the initial weight of the microcapsules, and $W_{2}$ represents the weight of the microcapsules after drying.

Solubility was determined according to the method described by Napiórkowska et al. (2024a). Briefly, 0.5 g of microcapsule powder was dispersed in 50 mL of distilled water at 25°C and stirred at 60 rpm for 30 min. The suspension was then centrifuged at 9000 × *g* for 5 min. Subsequently, 25 mL of the supernatant was transferred into preweighed Petri dishes and dried at 105°C for 24 h until constant weight was achieved. Solubility was calculated using Equation (6).

(6) $$\mathrm{Solubility}\;\;\left(\%\right)=\left(\mathrm{Dry}\;\mathrm{weight}\;\mathrm{of}\;\mathrm{the}\frac{\mathrm{solute}}{\mathrm{initial}}\mathrm{weight}\right)\;\times 100$$

#### 2.6.3 Bulk Density, Tapped Density, Carr's Index, and Hausner Ratio

Bulk density and TD were determined according to Napiórkowska et al. (2024b). Briefly, 1 g of powder was transferred into a 10 mL graduated cylinder and the occupied volume was recorded. TD was determined after manually tapping the cylinder under its own weight for 1 min and recording the final volume. BD and TD were calculated using Equations (7) and (8), respectively. CI and HR were calculated according to Mohammed et al. (2021) using Equations (9) and (10) to evaluate the flowability and cohesiveness of the microcapsules.

(7) $$\mathrm{BD}\;\;\left(g/cm^{3}\right)=\;\mathrm{Sample}\;\mathrm{weight}/\mathrm{Sample}\;\mathrm{volume}$$

(8) $$\mathrm{TD}\;\;\left(g/cm^{3}\right)=\;\mathrm{Sample}\;\mathrm{weight}/\mathrm{Tapped}\;\mathrm{volume}\;$$

(9) $$\mathrm{CI}\;\;\left(\%\right)=\left(\mathrm{TD}\;-\;\mathrm{BD}/\mathrm{TD}\right)\;\times \;100$$

(10) HR=TD/BD

#### 2.6.4 Crystallinity

The crystalline structures of the microcapsules and the wall materials were analyzed by XRD. XRD patterns were obtained using an X‐ray diffractometer (Bruker AXS D8 Advance, Madison, WI, USA) operated at 40 kV and 40 mA with Cu Kα radiation (*λ* = 1.540 Å). Diffraction patterns were recorded over a 2*θ* range of 5°–50° at a scanning rate of 10°/min at room temperature (25°C; Mu et al. 2024).

#### 2.6.5 Fourier Transform Infrared Spectroscopy Analysis

FTIR analysis was performed using the KBr pellet method in the range of 4000–400 cm^−^^1^ with a resolution of 4 cm^−^^1^ and 32 scans using a Nicolet iS50 spectrometer (Thermo Fisher Scientific, USA). The FTIR spectra of the wall materials, core oil, and microcapsules were recorded (Chen et al. 2025a).

#### 2.6.6 Thermogravimetric Analysis

To evaluate the thermal stability of the microcapsules, TGA analysis was performed using a thermogravimetric analyzer (TA Instruments SDT‐650, USA). The analysis was carried out over a temperature range of 30–600°C at a heating rate of 10°C/min under a nitrogen atmosphere (Mutlu 2023).

### 2.7 Antibacterial Activity of NSEO‐MCs

The antibacterial activity of the microcapsules was evaluated using a modified agar well diffusion method based on Plati and Paraskevopoulou (2023). *E. coli* and *S. aureus* were cultured in brain heart infusion broth and incubated at 37°C for 24 h. The cultures were then streaked onto Mueller–Hinton agar plates to obtain colonies. A bacterial suspension was prepared in sterile saline and adjusted to 0.5 McFarland standard. Subsequently, 0.1 mL of each bacterial suspension was spread on Mueller–Hinton agar plates. Wells (7 mm in diameter) were aseptically punched into the agar using a sterile cork borer. Dried microcapsules containing 10.0 mg of NSEO were accurately weighed and placed into the wells. The plates were incubated at 37°C for 24 h, and the diameters of the inhibition zones were measured in millimeters.

### 2.8 Statistical Analysis

For encapsulation optimization, statistical analyses were performed using Minitab software version 22.3 (Minitab LLC, State College, PA, USA). RSM based on a Box–Behnken design was applied, and the effects of pH, wall material concentration, and oil concentration, as well as their two‐way interactions, on EE were evaluated by ANOVA. The optimal conditions were predicted using the response optimizer function in Minitab.

Additional statistical analyses were performed using SPSS version 27 (IBM, Chicago, IL, USA). One‐way ANOVA followed by Tukey's post hoc test was used to determine statistical differences. A *p* value < 0.05 was considered statistically significant.

## 3 Results and Discussion

### 3.1 Optimizations of NSEO‐MCs by RSM

The experimental data for EE (%) were fitted to second‐order polynomial regression models, which describe the relationship between the response variable and the independent factors and are presented as follows (Equation 11):

(11) $$\begin{array}{ccc} \mathrm{EE}\left(\%\right) & = & -341.8+189.6A+72.2B-40.5C-23.51A2-29.91B2\\ & & -15.51C2+0.00AB+3.60AC+30.00BC \end{array}$$

where *A*, *B*, and *C* represent pH, wall material concentration, and oil concentration, respectively.

As seen in Table 2, the model was found to be statistically significant (*F* = 83.20, *p* < 0.001). High *R* ^2^ (99.34%), adjusted *R* ^2^ (98.14%), and predicted *R* ^2^ (95.24%) values indicate that the model explains the experimental data with high accuracy and also possesses strong predictive ability. Furthermore, nonsignificant lack‐of‐fit (*p* > 0.05) indicates that the model fits the data well and that the model errors are negligible.

**TABLE 2:** Analysis of variance (ANOVA) for the quadratic polynomial model of EE (%).

Source	DF	Sum of squares	Mean square	*F* value	*p* value	Significance
Model	9	1228.45	136.495	83.20	0.000	^***^
Linear	3	657.34	219.113	133.55	0.000	^***^
pH	1	52.02	52.020	31.71	0.002	^**^
Wall material concentration	1	312.50	312.500	190.47	0.000	^***^
Oil concentration	1	292.82	292.820	178.48	0.000	^***^
Square	3	342.87	114.292	69.66	0.000	^***^
pH^2^	1	127.59	127.587	77.77	0.000	^***^
(Wall material concentration)^2^	1	206.49	206.494	125.86	0.000	^***^
(Oil concentration)^2^	1	55.54	55.538	33.85	0.002	^**^
Two‐way interaction	3	228.24	76.080	46.37	0.000	^***^
pH × wall material concentration	1	0.00	0.000	0.00	1.000	NS
pH × oil concentration	1	3.24	3.240	1.97	0.219	NS
Wall material concentration × oil concentration	1	225.00	225.000	137.14	0.000	^***^
Error	5	8.20	1.641	—	—	—
Lack‐of‐fit	3	2.94	0.980	0.37	0.785	NS
Pure error	2	5.26	2.632	—	—	—
Total	14	1236.66	—	—	—	—
Model summary		***S* = 1.28088**, ***R* ^2^ = 99.34%, Adj *R* ^2^ = 98.14% Pred *R* ^2^ = 95.24%**				

*** *p* < 0.001.

** *p* < 0.01, NS, not significant (*p* > 0.05).

Linear, quadratic, and two‐way interaction terms were found to be significant (*p* < 0.05). Specifically, wall material concentration and oil concentration had the highest *F* values and were identified as the most influential factors in the response. pH was also statistically significant but had a weaker effect. In agreement with the present findings, previous studies have reported that parameters such as pH, wall material composition and concentration, and core‐to‐wall ratio play critical roles in complex coacervation systems, as they directly influence electrostatic interactions and the formation of stable microcapsule structures (Ma et al. 2024). The significance of the quadratic terms indicates that the system exhibits nonlinear behavior and that an optimum point exists. Among the interaction terms, only the interaction between wall material concentration and oil concentration was found to be significant, while the other interactions were not statistically significant (*p* > 0.05). It should be noted that the nonsignificance of pH‐related interaction terms does not contradict the visual trends observed in the response surface plots. In Box–Behnken designs, response surface plots are generated from the full second‐order polynomial model, which includes all terms regardless of their statistical significance. Consequently, even nonsignificant interaction terms contribute to the visual shape of the surface, and apparent trends may be observed in the plots without corresponding statistical significance. This is an inherent characteristic of RSM methodology rather than an inconsistency in the data. Furthermore, the significant linear effect of pH (*F* = 31.71, *p* = 0.002) confirms that pH exerts a meaningful independent influence on EE%, even in the absence of significant two‐way interactions with other variables.

**FIGURE 1:**
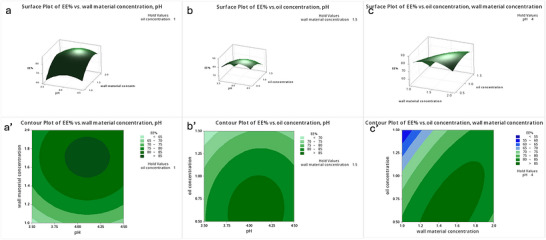
Response surface (a–c) and contour plots (a′–c′) showing the effect of pH, wall material concentration, and oil concentration on the EE (%) of microcapsules.

Although the interaction between pH and wall material concentration was not statistically significant (*p* > 0.05), the response surface plots (Figure 1a) indicate the presence of an optimum region. EE% reaches its maximum at moderate pH values (∼4) and higher wall material concentrations, while it decreases at lower pH and lower wall material concentrations. This suggests that complex formation is more effective under appropriate pH conditions, and increasing the wall material concentration enhances EE by providing better coverage of the core material. Although the protein–polysaccharide ratio was kept constant in this study, previous research has shown that both pH and biopolymer composition influence electrostatic interactions and coacervation behavior. This suggests that pH may also modulate the effectiveness of wall material concentration by affecting intermolecular interactions (Ayar‐Sumer et al. 2024). The observed increase in EE% with increasing wall material concentration at optimal pH can be attributed to enhanced electrostatic interactions between oppositely charged components. However, excessive polymer content may disrupt charge balance, reducing EE (Djihad et al. 2024).

Similarly, although no statistically significant interaction was observed between pH and oil concentration (*p* > 0.05), the response surface plots (Figure 1b) suggest a trend in EE%. At low oil concentrations, EE% remains relatively high across the pH range, whereas at higher oil concentrations, EE% decreases, particularly at nonoptimal pH values. This indicates that while moderate oil levels can be effectively encapsulated, excessive oil may reduce efficiency, especially when electrostatic interactions are not at their optimum. EE% increased up to an optimum oil concentration and decreased thereafter, which is consistent with previous studies reporting that excessive oil loading reduces EE due to insufficient wall material and increased surface oil formation (Hu et al. 2024; Pu et al. 2025).

As seen in Figure 1c,c’, the interaction between wall material concentration and oil concentration shows that EE% increases with increasing wall material concentration, particularly at moderate oil levels. At low wall material concentrations, increasing oil concentration leads to a marked decrease in EE%, indicating insufficient encapsulation capacity. In contrast, higher wall material concentrations can better accommodate increasing oil content, resulting in improved EE. However, at excessive oil concentrations, EE% decreases even at high wall material levels, suggesting that the system reaches its saturation limit. This behavior is consistent with previous studies reporting that excessive core material reduces the relative proportion of wall material, leading to thinner and less compact coating layers, increased surface oil content, and consequently lower EE (Hu et al. 2024).

The optimization analysis revealed that pH, wall material concentration, and oil concentration were critical factors for maximizing encapsulation efficiency (EE%). The response optimizer identified the optimum conditions at pH 4.09, wall material concentration of 1.52%, and oil concentration of 0.64%. Under these conditions, the model predicted an EE% value of 87.38%, with a composite desirability of 1.00. To validate the predictive accuracy of the model, a confirmatory experiment was conducted under the identified optimal conditions. The experimentally obtained EE was 87.82%, which was in close agreement with the predicted value of 87.38%, confirming the adequacy of the developed RSM model.

### 3.2 Characterization of Microcapsules

#### 3.2.1 Morphology

**FIGURE 2:**
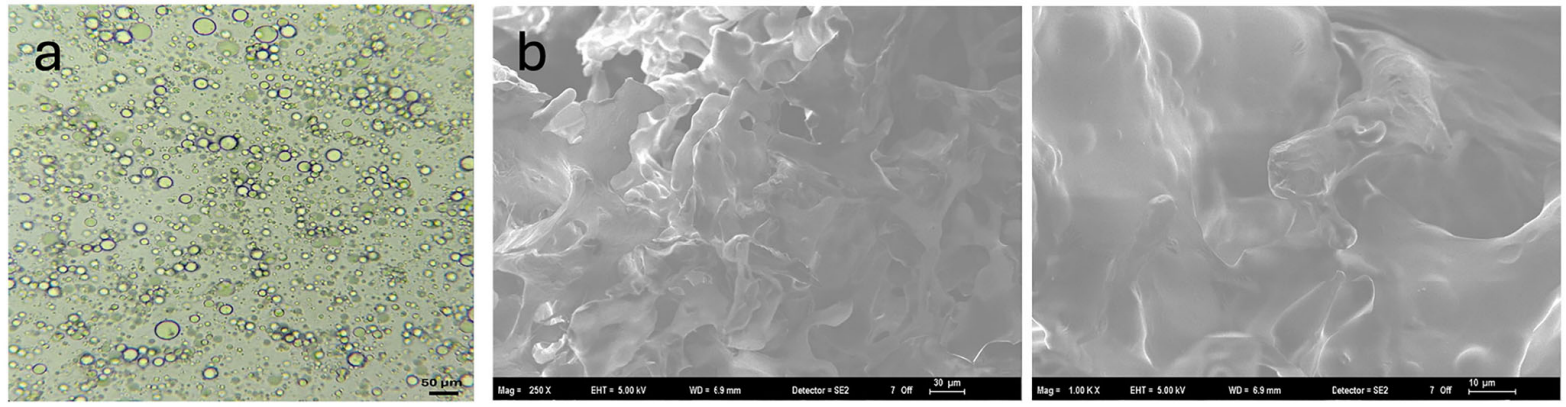
Microphotograph (×100) of liquid microcapsules (a) and SEM images of freeze‐dried microcapsules at ×250 and ×1000 magnification (b).

Optical microscopy was used to observe liquid microcapsules and SEM was conducted after freeze‐drying (Figure 2). Optical microscopy images (Table 3) showed that the microcapsules had a predominantly spherical morphology with a relatively broad size distribution, indicating a polydisperse system. These structures are likely formed through the initial generation of emulsion droplets, followed by complex coacervation between Ge and GA, which leads to the encapsulation of the oil phase. Similar behavior has been reported in Ge–GA system, where complex particles are formed as aggregates of emulsion droplets, and their size tends to increase with higher wall material and oil content (Sun et al. 2025).

**TABLE 3:** Physicochemical properties of freeze‐dried microcapsules.

Solubility (%)	Moisture content (%)	Hygroscopicity (%)	Bulk density (g/cm^3^)	Tapped density (g/cm^3^)	Carr's index	Hausner ratio
19.44	4.65	13.08	0.0889	0.1127	17.78	1.22

SEM images of the freeze‐dried samples revealed a very different morphology. The structures appeared irregular, aggregated, and partially collapsed, with rough and wrinkled surfaces. This change in morphology can be mainly attributed to the freeze‐drying process. During drying, rapid water removal likely causes structural shrinkage and leads to the formation of porous agglomerates, which explains the loss of the original spherical shape. Similar observations have been reported in previous studies, where freeze‐drying resulted in nonspherical and porous structures (Chen et al. 2023). This morphological transformation can be attributed to ice crystal formation during the freezing stage, which disrupts the original spherical architecture of the coacervate shell, followed by sublimation‐induced structural collapse during the drying stage.

Despite these structural changes, no clear signs of surface oil leakage or phase separation were observed, suggesting that the encapsulated phase remained well confined within the matrix. This indicates that the microcapsule structure was not severely disrupted during drying. This is further supported by the high EE (87.82%) obtained under optimized conditions, confirming that the integrity of the oil–wall interface was maintained throughout the freeze‐drying process. Overall, the results suggest that while spherical microcapsules are successfully formed in the liquid state, the final dried morphology is largely governed by the dehydration process, leading to more compact and irregular structures.

Although particle size distribution and zeta potential were not determined in this study, optical and electron microscopy observations confirmed that the microcapsules were formed at the micron scale, consistent with the expected size range for Ge–GA complex coacervates reported in the literature.

#### 3.2.2 Hygroscopicity, Moisture Content, and Water Solubility

The hygroscopicity of the microcapsules was 13.08 ± 1.21%, indicating a moderate tendency to absorb moisture from the surrounding environment. Hygroscopicity reflects the ability of a material to uptake atmospheric moisture, and high values may lead to lipid oxidation and powder aggregation, negatively affecting product quality (Bajac et al. 2022).

This behavior can be attributed to the hydrophilic nature of Ge and GA, which contain polar groups capable of interacting with water molecules. Similar results have been reported for Ge–GA systems (Chen et al. 2023). The hygroscopicity value obtained in the present study (13.08%) was higher than that reported by Djihad et al. for gelatin/carrageenan complex coacervation microcapsules (9.86%), which may be attributed to differences in wall material composition, as gum arabic contains a higher proportion of hydrophilic arabinogalactan fractions compared to carrageenan (Djihad et al. 2024). Furthermore, the complex coacervate wall formed by Ge–GA electrostatic interactions may reduce the number of available water‐binding sites on the polymer chains, thereby limiting moisture uptake compared to individual biopolymers (Bakry et al. 2019; Ng et al. 2022). The hygroscopicity value obtained in the present study is also consistent with the general range reported for freeze‐dried microcapsules with hydrophilic wall systems (Hashim et al. 2024).

The moisture content of the microcapsules was 4.65 ± 0.15%, indicating a relatively low amount of residual water. Moisture content is an important factor for powder stability, since high levels can promote microbial growth and negatively affect storage stability. In general, values around 4% are considered suitable for the storage and transportation of microcapsule powders (Wang et al. 2023).

The solubility of the microcapsules was found to be 19.44 ± 0.84%, indicating a limited level of dispersibility in aqueous systems. Solubility is an important parameter, as it reflects the ability of the powder to interact with water and influences its potential applications.

The relatively low solubility observed in this study may be related to the compact and aggregated structure of the microcapsules, which can restrict water penetration and slow down dissolution. In addition, solubility is known to be affected by factors such as polymer concentration and test conditions, particularly water temperature. Previous studies have reported higher solubility values when higher temperatures (e.g., 40°C) and increased polymer concentrations were used (Zuanon et al. 2013). Furthermore, the crosslinked nature of the Ge–GA coacervate wall, stabilized by electrostatic interactions between oppositely charged biopolymers, inherently limits water penetration into the matrix, contributing to the observed low solubility. The relatively low solubility observed here contrasts with higher values reported for spray‐dried microcapsules using carrageenan‐based wall systems (Bakry et al. 2019), which may be attributed to differences in drying method and wall material hydrophilicity. Specifically, freeze‐drying produces a more compact and less porous matrix compared to spray drying, restricting water access to the core material (Ng et al. 2022).

#### 3.2.3 Bulk Density, Tapped Density, Carr's Index, and Hausner Ratio

High BD and TD are generally preferred for powder handling, as they allow storage in smaller volumes and limit the presence of void spaces that may facilitate air penetration and subsequent lipid oxidation. These parameters are also used to evaluate the packing behavior and flow characteristics of powders (Kan et al. 2026; Mu et al. 2024). In the present study, however, the BD and TD values were found to be 0.0889 ± 0.00962 g/cm^3^ and 0.1127 ± 0.04887 g/cm^3^, respectively, indicating a relatively low‐density and more porous powder structure. This may be related to the porous nature of the particles formed during freeze‐drying, where higher pore volume has been reported compared to other drying methods. This behavior is also consistent with the SEM observations, which revealed a more open and less compact particle structure.

CI and HR are commonly used to evaluate powder flow behavior and are calculated from BD and TD values. A CI below 15% or a HR below 1.18 indicates good flowability, whereas a CI above 18% or a HR above 1.22 indicates poor flowability (Chen et al. 2025a). In the present study, the CI (17.78 ± 1.92%) and HR (1.2167 ± 0.02887) fall between these ranges, indicating moderate flowability and acceptable interparticle cohesion. These results show that the powder does not exhibit severe flow limitations despite its porous and low‐density structure. The CI and HR values obtained in this study are comparable to those reported for spray‐dried oil body microcapsules stabilized with maltodextrin (CI, 16.55%; HR: 1.20; Zhu et al. 2022), and notably lower than those reported for freeze‐dried essential oil microcapsules prepared with whey protein and berry wax wall systems (CI, 23.05%–39.38%; HR: 1.30–1.65; Hashim et al. 2024), indicating that the Ge–GA coacervate system provides acceptable powder flow properties regardless of the drying‐induced porous structure.

#### 3.2.4 Crystallinity

**FIGURE 3:**
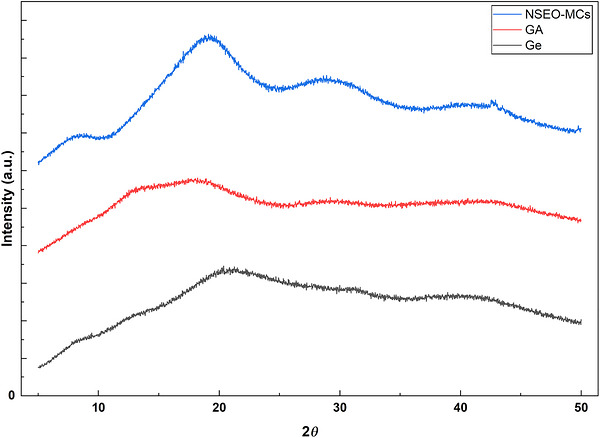
XRD patterns of Ge, GA, and NSEO‐MCs.

The XRD patterns of Ge, GA, and NSEO‐MCs are presented in Figure 3. Pure Ge exhibited a broad diffraction peak centered at approximately 2*θ* = 20°, which is characteristic of its semicrystalline nature arising from the α‐helix and triple‐helical molecular arrangement. GA displayed a broad amorphous diffraction pattern with a weak, ill‐defined maximum at approximately 2*θ* ≈ 19°, consistent with the disordered polysaccharide chain structure. In the NSEO‐MCs diffractogram, the broad peak at approximately 2*θ* = 20° was retained but showed a marked increase in intensity compared to either individual biopolymer. No sharp crystalline peaks attributable to free NSEO were detected throughout the measured 2*θ* range, indicating that the NSEO was successfully encapsulated within the polymeric matrix in a noncrystalline state.

The observed increase in peak intensity at 2*θ* ≈ 20° in NSEO‐MCs relative to the individual components can be attributed to the formation of a more compact and densely packed amorphous matrix resulting from the electrostatic interactions between positively charged Ge and negatively charged GA during complex coacervation. As previously reported by Eltarahony et al., the broadness and intensity of the diffraction peak in Ge–GA composite systems increases with increasing GA incorporation, which was attributed to enhanced intermolecular interactions and greater structural organization within the amorphous network (Eltarahony et al. 2025). Given that Ge and GA exhibit overlapping broad diffraction peaks in the 2*θ* ≈ 19°–20° region, their simultaneous presence in the coacervate wall is expected to produce a reinforced scattering signal, consistent with what was observed in the NSEO‐MCs pattern. Taken together, the XRD results confirm that complex coacervation between Ge and GA produced a structurally integrated amorphous wall matrix, within which NSEO was effectively entrapped. The overall amorphous character of NSEO‐MCs is consistent with previous studies reporting similar XRD profiles for essential oil microcapsules prepared by complex coacervation (Yang et al. 2024).

#### 3.2.5 Fourier Transform Infrared Analysis

**FIGURE 4:**
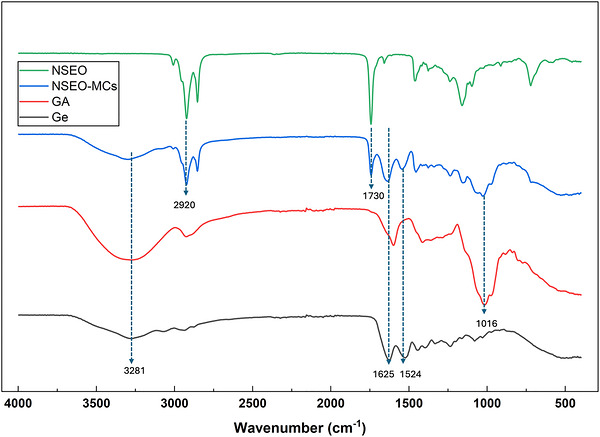
FTIR spectra of NSEO, Ge, GA, and NSEO‐MCs.

The FTIR spectra of Ge, GA, NSEO, and NSEO‐MCs are presented in Figure 4. The spectrum of Ge displayed characteristic protein bands: a broad N–H/O–H stretching band at ∼3300 cm^−^^1^, and Amide I (∼1630 cm^−^^1^) and Amide II (∼1530 cm^−^^1^) bands arising from C═O stretching and N–H bending of peptide bonds, respectively (Devi et al. 2023). GA exhibited a broad O–H stretching band at ∼3300 cm^−^^1^, a carboxylate (COO^−^) asymmetric stretching band at ∼1600 cm^−^^1^, and strong C─O─C glycosidic linkage vibrations in the 1000–1100 cm^−^^1^ region, consistent with its polysaccharide structure. The NSEO spectrum was dominated by two intense C─H stretching bands at ∼2920 and ∼2850 cm^−^^1^, characteristic of the aliphatic methyl and isopropyl substituents of its terpenoid components, along with a C═O stretching band at ∼1730 cm^−^^1^ and a band at ∼1630 cm^−^^1^ attributed to C═O stretching of thymoquinone (Bhavikatti et al. 2024). In the NSEO‐MCs spectrum, the C═O stretching band at ∼1730 cm^−^^1^ remained detectable, albeit with reduced intensity, confirming that the chemical integrity of NSEO constituents was preserved within the Ge–GA matrix without covalent modification. This finding is consistent with the antimicrobial results in Section 3.2.7, where encapsulation did not reduce the inhibitory activity of NSEO.

In the NSEO‐MCs spectrum, the characteristic Amide I and Amide II bands of Ge were retained, and the C─O─C bands of GA remained visible in the fingerprint region, confirming the presence of both wall materials in the capsule structure. Notably, the intense C─H stretching bands of NSEO at ∼2920 and ∼2850 cm^−^^1^ showed a marked attenuation in the NSEO‐MCs spectrum relative to free NSEO, indicating that the essential oil was successfully entrapped within the Ge–GA coacervate wall matrix (Yang et al. 2024). This observation confirms that the components of NSEO‐MCs are not simply physically mixed, but that NSEO is structurally enclosed within the polymeric wall.

A change in the Amide I band profile in the NSEO‐MCs spectrum, including an increase in band intensity and sharpness compared to pure Ge, suggests conformational modifications in the gelatin backbone upon interaction with GA during coacervation. Such changes in the Amide I region are indicative of electrostatic interactions between the carboxyl groups of GA and the amide groups of Ge (Derkach et al. 2020, 2022).

#### 3.2.6 Thermogravimetric Analysis

**FIGURE 5:**
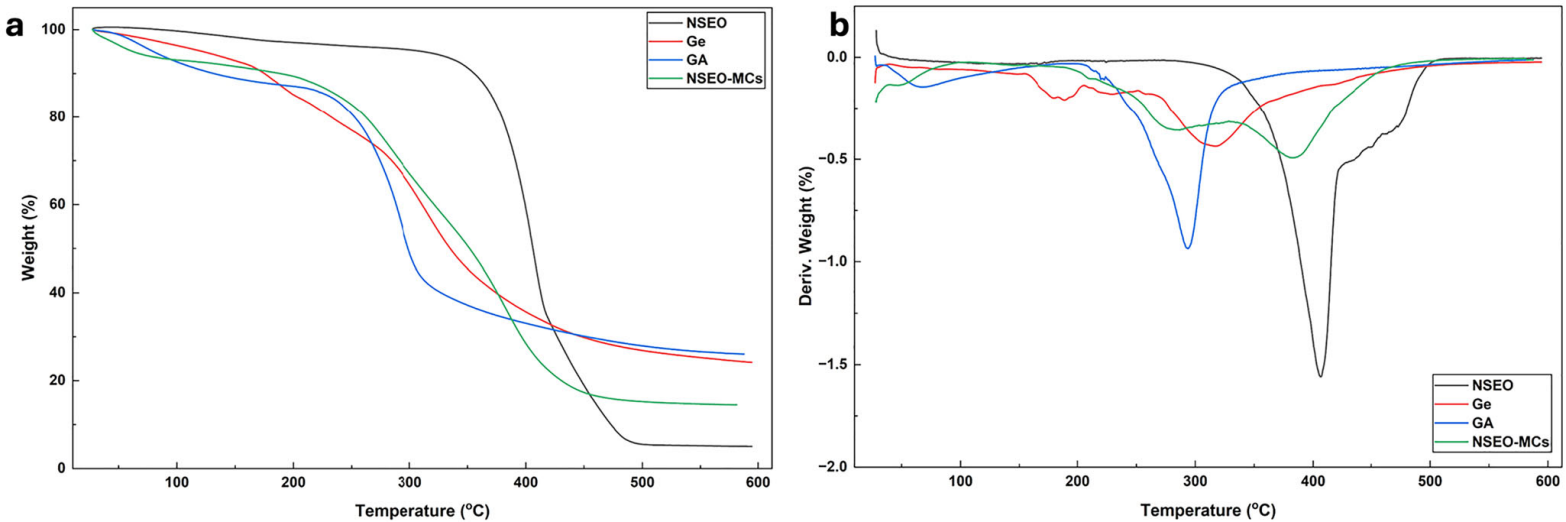
TGA (a) and DTG (b) curves of NSEO, Ge, GA, and NSEO‐MCs.

The thermal degradation behavior of Ge, GA, NSEO, and NSEO‐MCs was investigated by TGA and DTG analyses, as shown in Figure 5a,b. Free NSEO exhibited high thermal stability up to approximately 300°C, retaining ∼95% of its initial mass, followed by a rapid and near‐complete degradation between 300 and 480°C, with a sharp DTG peak at ∼410°C and a residual mass of approximately 5% at 600°C. Ge showed a gradual, multistage degradation beginning with moisture loss below 150°C (∼5%–8% mass loss), followed by a broad main degradation stage between 200 and 420°C associated with peptide bond cleavage and protein backbone decomposition, leaving ∼25% residue at 600°C. GA exhibited an early onset of degradation from ∼100°C, with its most rapid mass loss occurring between 200 and 350°C, as confirmed by a pronounced DTG minimum at ∼290°C, and a residual mass of ∼26% at 600°C.

The DTG peak of NSEO‐MCs shifted from approximately 410°C (free NSEO) to approximately 450–460°C, representing a shift of ∼40–50°C, while becoming considerably broader and of lower intensity. This thermal stabilization indicates that the Ge–GA coacervate wall effectively delayed volatilization of the entrapped oil by acting as a physical diffusion barrier. The broadening of the DTG peak confirms that NSEO was homogeneously distributed within the polymeric matrix rather than existing as a discrete free phase, requiring more energy for complete release (Liu et al. 2025; Yang et al. 2023). This degree of thermal protection is consistent with previous reports on Ge–GA complex coacervation systems (Li et al. 2022; Yang et al. 2024). This interpretation is further corroborated by the FTIR evidence presented in Section 3.2.5, where retention of NSEO‐characteristic bands confirmed physical entrapment without chemical modification of the oil constituents.

#### 3.2.7 In Vitro Antimicrobial Effects of Microcapsules

The inhibition zone diameters obtained for both NSEO‐MCs and free NSEO at all tested concentrations are presented in Table 4. The antimicrobial activity of NSEO‐MCs and free NSEO was evaluated against *E. coli* ATCC 25922 and *S. aureus* ATCC 29213 using the agar well diffusion method. Both forms exhibited dose‐dependent inhibitory activity, with inhibition zone diameters increasing significantly from 2.5 to 10 mg (*p* < 0.05). The antimicrobial properties of NSEO are primarily attributed to thymoquinone and other phenolic constituents, which disrupt bacterial cell membranes, increase ion permeability, and impair energy metabolism (Hyldgaard et al. 2012; Zhao et al. 2023).

**TABLE 4:** Inhibition zone diameters (mm) of NSEO‐MCs and free NSEO against *E. coli* and *S. aureus* at different concentrations.

Concentration	Sample	Inhibition zone diameter (mm)	
		*E. coli*	*S. aureus*
2.5 mg	NSEO‐MCs	7.33 ± 0.15 ^c^	7.77 ± 0.25 ^b^
	Free NSEO	7.37 ± 0.40 ^c^	8.13 ± 0.40 ^b^
5 mg	NSEO‐MCs	7.87 ± 0.31 ^bc^	8.40 ± 0.17 ^ab^
	Free NSEO	7.87 ± 0.23 ^bc^	8.43 ± 0.06 ^ab^
10 mg	NSEO‐MCs	8.67 ± 0.15 ^a^	9.00 ± 0.20 ^a^
	Free NSEO	8.60 ± 0.36 ^ab^	9.03 ± 0.25 ^a^

Values are expressed as mean ± SD (*n* = 3). Inhibition zone diameters are exclusive of the well diameter (7 mm). Different superscript letters within the same column indicate significant differences among all groups (*p* < 0.05).

*S. aureus* showed greater susceptibility than *E. coli* at all tested concentrations. This difference is well‐documented for hydrophobic essential oil constituents and is attributed to structural differences in the bacterial cell wall: gram‐positive bacteria possess a thick peptidoglycan layer without an outer lipopolysaccharide membrane, facilitating penetration of hydrophobic compounds, whereas the outer membrane of gram‐negative bacteria acts as an additional permeability barrier limiting diffusion (Hyldgaard et al. 2012; Koç et al. 2026; Mukurumbira et al. 2022).

No statistically significant difference was observed between NSEO‐MCs and free NSEO at any tested concentration (*p* > 0.05), demonstrating that the Ge–GA wall matrix did not reduce the antimicrobial efficacy of NSEO. This finding is consistent with Koç et al. (2026), who reported that clove oil microcapsules maintained or slightly improved antibacterial activity compared to free oil, particularly at higher concentrations, attributing this behavior to improved stability of volatile compounds, increased solubility and dispersion in aqueous media, and enhanced bioavailability of active constituents (Koç et al. 2026). Similarly, Plati and Paraskevopoulou demonstrated that oregano essential oil encapsulated in Ge–GA complex coacervates maintained antimicrobial properties against *E. coli* and *S. aureus*, with encapsulated samples releasing the oil rapidly into the agar medium during incubation (Plati and Paraskevopoulou 2023). Mukurumbira et al. further noted that complex coacervation generally preserves or enhances antimicrobial activity through physical and chemical stabilization of active compounds within the wall matrix (Mukurumbira et al. 2022). The preservation of antimicrobial efficacy in the present study confirms that the Ge–GA coacervate system is biocompatible with NSEO's active constituents and does not form interactions that compromise their biological activity (Zhao et al. 2023).

These findings are further supported by the FTIR and TGA results obtained in this study. FTIR analysis confirmed that NSEO was physically entrapped within the Ge–GA matrix through noncovalent interactions, with no evidence of covalent bond formation between the wall materials and the active constituents of NSEO. This physical encapsulation mechanism ensures that the bioactive compounds, particularly thymoquinone, retain their chemical integrity and remain available for antimicrobial action upon release. Furthermore, the enhanced thermal stability demonstrated by TGA reflects the formation of a compact and structurally integrated coacervate wall, which protects NSEO during processing and storage without chemically modifying its active components. Collectively, the FTIR, TGA, and antimicrobial results consistently indicate that the Ge–GA complex coacervation system acts as a physical protective barrier rather than a chemically reactive matrix, thereby preserving the full bioactive potential of NSEO.

## 4 Conclusion

This study demonstrated that NSEO can be successfully microencapsulated via Ge–GA complex coacervation under RSM‐optimized conditions (pH 4.09, wall material concentration 1.52%, oil concentration 0.64%), yielding an EE of 87.38%, which was experimentally validated (87.82%). Wall material concentration and oil concentration were the dominant factors governing EE%, while pH exerted a significant independent effect.

Characterization results revealed a consistent picture across multiple analytical levels. XRD confirmed amorphous entrapment of NSEO within the Ge–GA matrix. FTIR provided converging evidence of successful physical encapsulation, noncovalent Ge–GA interactions, and preservation of NSEO's chemical integrity—the latter confirmed by retention of the C═O band at ∼1730 cm^−^^1^. TGA demonstrated enhanced thermal stability of the encapsulated oil, consistent with its distribution within the polymeric matrix. Physicochemical properties including moisture content, hygroscopicity, and flowability were acceptable for food applications.

Antimicrobial evaluation showed dose‐dependent inhibitory activity against *E. coli* and *S. aureus*, with no significant difference between NSEO‐MCs and free NSEO, confirming that the Ge–GA system acts as a physical protective barrier that fully preserves the bioactive potential of NSEO. Future work should address colloidal characterization of the liquid coacervate phase, in vitro release profiling, oxidative stability during storage, and encapsulation of other hydrophobic bioactive compounds.

## Author Contributions

**Neslihan Mutlu**: conceptualization, methodology, investigation, writing – original draft, supervision. **Ümmü Gülsüm Koç**: investigation, formal analysis, writing – review and editing.

## Funding

The authors received no specific funding for this work.

## Conflicts of Interest

The authors declare no conflicts of interest.

## Data Availability

Data will be made available upon request.
